# Supplementary material for: Evaluating Methods of Updating Training Data in Long-Term Genomewide Selection
Source: G3 (Bethesda). 2017 Mar 15;7(5):1499–510. doi: 10.1534/g3.117.040550 (PMC5427505; doi:10.1534/g3.117.040550)
Supplement: Supplementary file 1 [file 1499file002.docx]

**Supplementary Table 1. Prediction Accuracy Across Breeding Cycles**

|  |  | Breeding Cycle | | | | | | | | | | | | | | |
| --- | --- | --- | --- | --- | --- | --- | --- | --- | --- | --- | --- | --- | --- | --- | --- | --- |
| Scenario | Method | 1 | 2 | 3 | 4 | 5 | 6 | 7 | 8 | 9 | 10 | 11 | 12 | 13 | 14 | 15 |
| Cumulative | Top | 0.722 (0.716, 0.728) | 0.566 (0.544, 0.588) | 0.51 (0.488, 0.532) | 0.469 (0.448, 0.49) | 0.423 (0.404, 0.441) | 0.399 (0.381, 0.417) | 0.354 (0.336, 0.373) | 0.326 (0.309, 0.342) | 0.31 (0.294, 0.327) | 0.258 (0.24, 0.276) | 0.245 (0.229, 0.261) | 0.21 (0.194, 0.226) | 0.194 (0.177, 0.211) | 0.157 (0.14, 0.174) | 0.147 (0.13, 0.164) |
| Cumulative | CDmean | 0.712 (0.705, 0.72) | 0.525 (0.51, 0.541) | 0.468 (0.449, 0.487) | 0.438 (0.421, 0.456) | 0.412 (0.394, 0.43) | 0.374 (0.358, 0.39) | 0.339 (0.323, 0.355) | 0.305 (0.29, 0.321) | 0.275 (0.259, 0.291) | 0.243 (0.227, 0.259) | 0.23 (0.215, 0.246) | 0.198 (0.183, 0.212) | 0.188 (0.173, 0.202) | 0.171 (0.157, 0.186) | 0.145 (0.13, 0.161) |
| Cumulative | No Change | 0.72 (0.712, 0.727) | 0.543 (0.533, 0.554) | 0.443 (0.432, 0.454) | 0.37 (0.36, 0.38) | 0.31 (0.3, 0.319) | 0.26 (0.251, 0.269) | 0.211 (0.201, 0.22) | 0.183 (0.174, 0.192) | 0.156 (0.146, 0.165) | 0.126 (0.116, 0.135) | 0.103 (0.094, 0.113) | 0.083 (0.073, 0.092) | 0.069 (0.06, 0.078) | 0.058 (0.049, 0.067) | 0.047 (0.037, 0.057) |
| Cumulative | PEVmean | 0.724 (0.716, 0.731) | 0.531 (0.515, 0.548) | 0.446 (0.424, 0.469) | 0.424 (0.405, 0.443) | 0.4 (0.382, 0.418) | 0.368 (0.352, 0.384) | 0.336 (0.32, 0.352) | 0.31 (0.296, 0.325) | 0.278 (0.261, 0.295) | 0.254 (0.238, 0.27) | 0.236 (0.221, 0.251) | 0.206 (0.191, 0.222) | 0.196 (0.183, 0.209) | 0.167 (0.153, 0.182) | 0.142 (0.129, 0.155) |
| Cumulative | Random | 0.715 (0.708, 0.722) | 0.539 (0.526, 0.552) | 0.474 (0.455, 0.493) | 0.434 (0.417, 0.452) | 0.392 (0.372, 0.412) | 0.37 (0.354, 0.387) | 0.339 (0.322, 0.356) | 0.303 (0.286, 0.32) | 0.271 (0.256, 0.286) | 0.233 (0.214, 0.252) | 0.214 (0.197, 0.23) | 0.198 (0.182, 0.215) | 0.183 (0.169, 0.197) | 0.159 (0.143, 0.176) | 0.149 (0.133, 0.165) |
| Cumulative | Tails | 0.717 (0.71, 0.724) | 0.551 (0.533, 0.568) | 0.497 (0.48, 0.514) | 0.475 (0.46, 0.491) | 0.438 (0.421, 0.454) | 0.414 (0.398, 0.43) | 0.375 (0.36, 0.391) | 0.346 (0.329, 0.363) | 0.315 (0.3, 0.33) | 0.276 (0.26, 0.293) | 0.236 (0.219, 0.253) | 0.219 (0.204, 0.235) | 0.207 (0.191, 0.223) | 0.171 (0.157, 0.186) | 0.161 (0.146, 0.176) |
| Cumulative | Bottom | 0.714 (0.707, 0.722) | 0.495 (0.478, 0.511) | 0.442 (0.424, 0.459) | 0.392 (0.376, 0.408) | 0.37 (0.355, 0.385) | 0.329 (0.313, 0.345) | 0.293 (0.278, 0.308) | 0.263 (0.247, 0.279) | 0.231 (0.215, 0.248) | 0.215 (0.199, 0.231) | 0.196 (0.18, 0.211) | 0.186 (0.171, 0.202) | 0.17 (0.156, 0.183) | 0.139 (0.125, 0.153) | 0.143 (0.13, 0.155) |
| Window | Top | 0.719 (0.712, 0.726) | 0.545 (0.518, 0.572) | 0.503 (0.481, 0.525) | 0.457 (0.436, 0.479) | 0.419 (0.399, 0.44) | 0.385 (0.366, 0.405) | 0.344 (0.323, 0.366) | 0.319 (0.298, 0.339) | 0.29 (0.271, 0.309) | 0.272 (0.253, 0.29) | 0.226 (0.205, 0.247) | 0.217 (0.198, 0.236) | 0.201 (0.181, 0.22) | 0.181 (0.162, 0.199) | 0.181 (0.162, 0.199) |
| Window | CDmean | 0.716 (0.709, 0.723) | 0.499 (0.482, 0.516) | 0.448 (0.429, 0.467) | 0.412 (0.395, 0.429) | 0.374 (0.358, 0.391) | 0.345 (0.327, 0.362) | 0.317 (0.299, 0.334) | 0.304 (0.286, 0.321) | 0.283 (0.265, 0.3) | 0.257 (0.239, 0.274) | 0.243 (0.225, 0.26) | 0.223 (0.204, 0.241) | 0.195 (0.178, 0.212) | 0.176 (0.16, 0.192) | 0.156 (0.139, 0.172) |
| Window | No Change | 0.717 (0.71, 0.725) | 0.543 (0.533, 0.553) | 0.438 (0.427, 0.448) | 0.363 (0.353, 0.374) | 0.296 (0.285, 0.306) | 0.246 (0.236, 0.256) | 0.203 (0.193, 0.212) | 0.171 (0.162, 0.181) | 0.142 (0.132, 0.152) | 0.117 (0.108, 0.126) | 0.1 (0.09, 0.11) | 0.086 (0.077, 0.095) | 0.069 (0.059, 0.079) | 0.062 (0.053, 0.071) | 0.054 (0.045, 0.063) |
| Window | PEVmean | 0.719 (0.713, 0.726) | 0.517 (0.503, 0.532) | 0.443 (0.424, 0.463) | 0.424 (0.408, 0.44) | 0.378 (0.363, 0.393) | 0.366 (0.348, 0.383) | 0.326 (0.307, 0.345) | 0.303 (0.285, 0.321) | 0.289 (0.271, 0.306) | 0.26 (0.243, 0.276) | 0.238 (0.22, 0.256) | 0.218 (0.201, 0.235) | 0.196 (0.179, 0.214) | 0.181 (0.165, 0.197) | 0.182 (0.167, 0.196) |
| Window | Random | 0.711 (0.704, 0.718) | 0.505 (0.488, 0.521) | 0.445 (0.426, 0.464) | 0.411 (0.394, 0.427) | 0.375 (0.358, 0.392) | 0.353 (0.337, 0.37) | 0.328 (0.311, 0.346) | 0.312 (0.295, 0.329) | 0.273 (0.255, 0.291) | 0.25 (0.232, 0.268) | 0.233 (0.216, 0.25) | 0.22 (0.202, 0.237) | 0.195 (0.177, 0.212) | 0.17 (0.153, 0.187) | 0.168 (0.151, 0.185) |
| Window | Tails | 0.722 (0.715, 0.73) | 0.538 (0.521, 0.555) | 0.499 (0.482, 0.516) | 0.461 (0.446, 0.477) | 0.424 (0.41, 0.439) | 0.385 (0.367, 0.403) | 0.367 (0.351, 0.382) | 0.348 (0.333, 0.364) | 0.305 (0.288, 0.322) | 0.292 (0.275, 0.309) | 0.27 (0.253, 0.287) | 0.232 (0.215, 0.25) | 0.216 (0.199, 0.233) | 0.188 (0.171, 0.206) | 0.158 (0.141, 0.175) |
| Window | Bottom | 0.715 (0.708, 0.721) | 0.459 (0.442, 0.476) | 0.41 (0.391, 0.428) | 0.357 (0.339, 0.375) | 0.338 (0.322, 0.354) | 0.327 (0.312, 0.343) | 0.306 (0.291, 0.322) | 0.272 (0.255, 0.289) | 0.254 (0.239, 0.27) | 0.244 (0.228, 0.261) | 0.229 (0.213, 0.244) | 0.208 (0.192, 0.224) | 0.19 (0.175, 0.206) | 0.173 (0.158, 0.188) | 0.177 (0.161, 0.192) |

Prediction accuracy was measured as the correlation between the predicted genotypic values of the selection candidates and the true genotypic values of the selection candidates. Accuracy was measured in each breeding cycle for each updating method within each updating scenario. Average accuracy is displayed with a 95% confidence interval in parentheses.
